# Clinical outcomes and urodynamic analysis of microsurgery combined with multilayer fascial tissue coverage for new urethra in the treatment of hypospadias in male children

**DOI:** 10.3389/fsurg.2025.1668520

**Published:** 2025-11-21

**Authors:** Wei Zheng, Shilei Guo, Xiaoqing Shi, Jie Wang, Bosong Zhang, Liwei Li, Chuang Liu, Bin Yang

**Affiliations:** Department of Urology Surgery, Baoding Hospital, Beijing Children’s Hospital Affiliated to Capital Medical University, Baoding, Hebei, China

**Keywords:** covering by multilayer fascial tissue coverage tissues, children, hypospadias, microsurgery, clinical effect

## Abstract

**Objective:**

To compare and analyze differences in the clinical effect and postoperative urodynamic indexes of microsurgery combined with covering new urethra by multilayer fascial tissue coverage tissues and conventional surgery alone in the treatment of male children with hypospadias.

**Methods:**

A total of 80 male children with penile hypospadias who were hospitalized in Baoding Hospital, Beijing Children's Hospital Affiliated to Capital Medical University were randomly divided into two groups, with 40 in each group. Children in the study group underwent microsurgery combined with covering new urethra by multilayer fascial tissue coverage tissues, while those in the control group were provided with traditional tubularized incised plate (TIP) urethroplasty alone. Further comparative analysis was performed on the operation effect, operation time, total intraoperative bleeding, postoperative length of stay in the hospital and the incidence of surgical complications between the two groups. All male children were followed up for 6 months to compare and analyze the changes of urodynamic parameters such as maximum urinary flow rate (*Q*_max_), mean urinary flow rate (*Q*_avc_), post-void residual urine (PVR) before and after surgery.

**Results:**

There were statistically significant differences that the operation time (*p* = 0.03) was longer while the postoperative length of stay in the hospital (*p* = 0.000) was shorter in the study group than those in the control group. The effective rate of the study group was 97.50%, while that of the control group was 82.50%, with statistically significant difference (*p* = 0.025). The incidence of surgical complications was 7.50% in the study group and 25.00% in the control group 3 months after operation (*p* = 0.034). There was statistically significant difference that the HOSE score of the study group was significantly higher than that of the control group (*p* = 0.000). Meanwhile, there was no significant difference in indicators such as *Q*_max_, *Q*_avc_ and PVR between the two groups before surgery; while the levels of *Q*_max_ and *Q*_avc_ in the study group were higher than those in the control group 6 months after surgery, with statistically significant difference (*p* = 0.000).

**Conclusion:**

Microsurgery combined with covering new urethra by multilayer fascial tissue coverage tissues has certain clinical value in the treatment of male children with hypospadias, which shows good therapeutic effect, shorter length of stay in the hospital, lower incidence of postoperative complications, and significantly improved urodynamic indicators, despite slightly complicated operation and relatively longer duration of operation.

## Introduction

Hypospadias has been recognized to be a common congenital malformation in Pediatric Urology. Children with hypospadias generally show ectopic position of the urethral orifice, chordee of penis and scarf-like changes in the dorsal foreskin of the penis (1). It is a highly heterogeneous multifactorial disease, which may be attributed to multiple genetic and environmental factors (2). Hypospadias is commonly treated by surgery to restore its appearance and function. It can be corrected at any age, without significant difference in the risk of complications, functional recovery and cosmetic effect. However, correction at an earlier stage is recommended by most researchers (3). At present, there are many corrective operations for hypospadias, yet accompanied by relatively higher incidence of postoperative complications. The major surgical complications are postoperative urinary fistula, urethral stricture, and unsatisfactory appearance, with the highest rate of urinary fistula in particular (4). Therefore, great concern in the treatment of hypospadias has been attached to the exploration of appropriate approaches to reduce the surgical complications.

The construction of abundant tissue barriers between the newly established urethra and the skin is the key to the surgery for hypospadias, which can reduce the occurrence of postoperative urinary fistula and promote the healthy formation of the newly formed urethra (5).

With the continuous development of science and technology, initial success has been realized in the application of microscopy for the repair of hypospadias. Through microscopic operation, the direction of blood vessels can be clearly visualized to ensure good blood supply of the covered tissue, leading to an improved success rate of surgery and hence reduced incidence of postoperative complications (6). With respect to the above, this study explored the combination of multilayer fascial tissue coverage tissue to cover the new urethra and microsurgery to improve the surgery for hypospadias, so as to reduce the occurrence of postoperative complications, which are reported as follows:

## Materials and methods

### Study population

**Figure 1:**
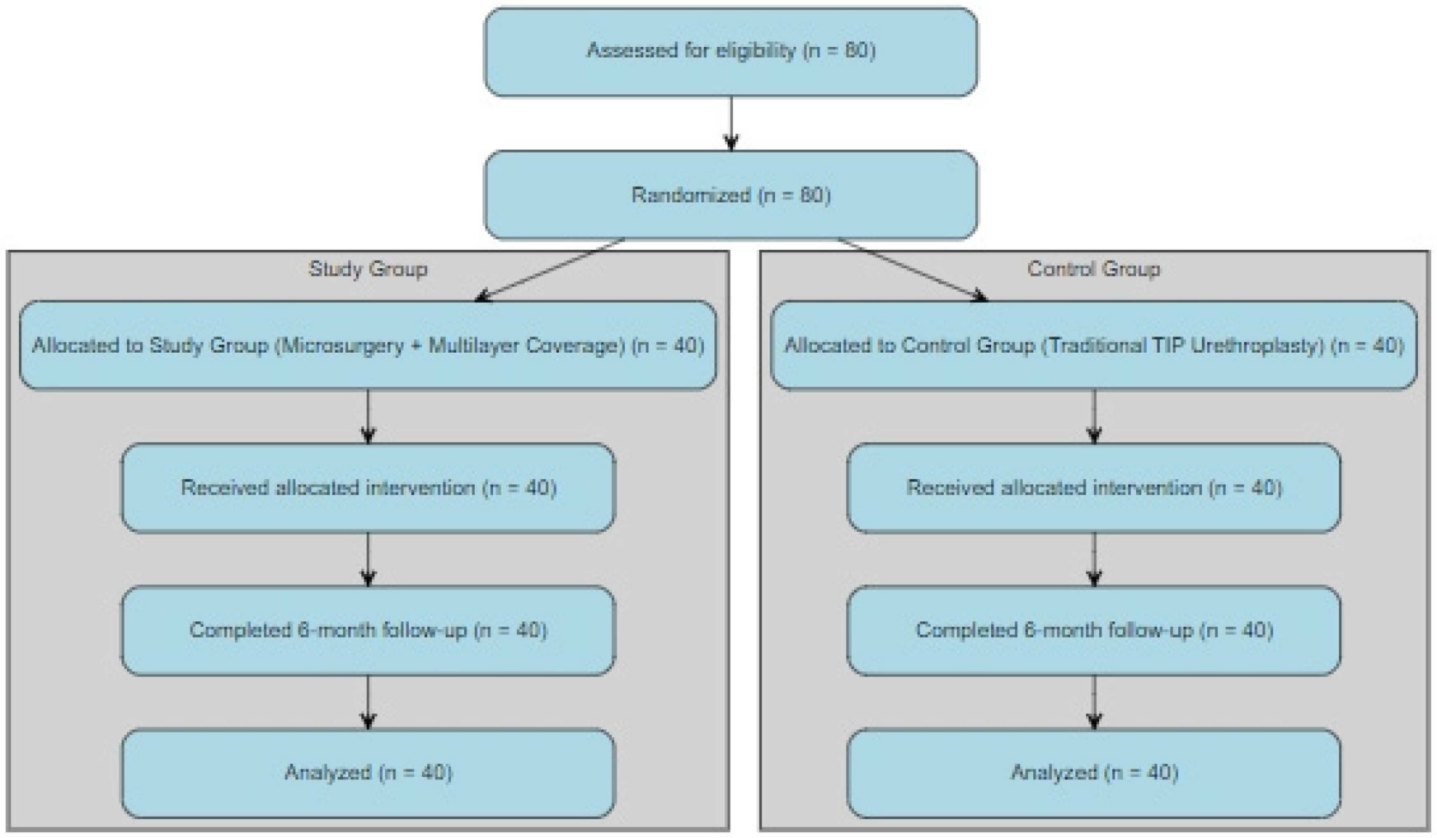
Research flowchart.

This study is a prospective randomized controlled trial, in which children were assigned to the study group or the control group using a random number table. A total of 80 male children with penile hypospadias who were hospitalized in Baoding Hospital, Beijing Children's Hospital Affiliated to Capital Medical University were enrolled Children in the study group underwent microsurgery combined with covering new urethra by multilayer fascial tissue coverage tissues, while those in the control group were provided with traditional urethroplasty (Figure 1). The study was approved by the Institutional Ethics Committee of Baoding Hospital, Beijing Children's Hospital Affiliated to Capital Medical University [No. 2021(10); date: October 27th, 2021].

Inclusion criteria: ① male children who met the diagnostic criteria for penile hypospadias (7); ② male children without obvious chordee of penis or with mild chordee of penis that required no surgical correction; ③ male children with flat urethral plates that were ≥6 mm in width and had good elasticity (Children with distal hypospadias and a urethral plate width of ≥6 mm were selected to control for anatomical heterogeneity and facilitate comparison of the clinical outcomes of different surgical approaches); ④ male children with urethral orifice located at the distal end and in the middle of the penis; ⑤ male children who aged ≤12 years old; ⑥ male children without heart, lung and other important organ diseases and other surgical contraindications; ⑦ male children with complete clinical data; ⑧ male children whose family members agreed to accept the study and signed the informed consent form; and ⑨ male children without obvious symptoms of mental nervous system, with good treatment compliance, and those who could cooperate with the study.

Exclusion criteria: ① male children with chordee of penis requiring surgical correction; ② male children with secondary surgical intervention; ③ male children with urethral orifice at the junction of penis and scrotum; ④ male children with severe liver and kidney dysfunction who cannot tolerate surgery; ⑤ male children with unsatisfied control of local infection; and ⑥ male children who were unable to cooperate to complete the study.

### Surgical methods

No preoperative hormonal treatment was administered. Ischemia time was recorded during surgery. The study group performed the operation using a microscope with 10x magnification, while the control group did not use a microscope. Children in the control group were treated with traditional tubularized incised plate (TIP) urethroplasty. In terms of the specific surgical procedures, under general anesthesia, male children were kept in their supine position and given routine disinfection of perineum and surgical towel laying, suture with 4–0 threads to fix the penis head combined with proper traction. After measuring the distance between the urethral orifice and the penis head with a ruler, a U-shaped incision was made under the urethral orifice to bypass around the urethral orifice and extend to the penis head in parallel on both sides. A circumferential incision of the foreskin was further made 0.5 cm below the coronary sulcus to Buck fascia to release the skin and subcutaneous fascia, and straighten the penis. After indwelling the urethral catheter, 4–0 absorbable thread was used for intermittent evting suture of the marginal flap of the urethral plate without tension. Then, the pedicled sarcolemma flap on the dorsal side of the penis was dissociated to cover the newly constructed urethra, followed by median longitudinal incision of the dorsal foreskin to the ventral side to transfer the flap to wrap the penis. The multilayer fascial tissue coverage includes the subcutaneous fascia, dartos fascia, and other layers, which are applied successively to enhance the blood supply to the urethra and its resistance to infection. After that, the incision was closed and dressed with sterile gauze.

The operation of the study group was performed under the microscope intraoperatively, using 6–0 Vicryl for suture. As for the procedure steps, on the basis of the control group, the superficial fascia of the ventral penis in the 4–8 O'clock direction was sharply separated from the proximal part of the penis to the mid-distal part of the penis with a microsurgical scissors until it was about 3 mm below the original urethral orifice. Then, the superficial fascia of the penis was turned up 180° to form a tipping bucket-shaped sarcolemma flap, which was then sutured to the urethra covered with sarcolemma on the dorsal side of the penis using 6–0 Vicryl to form a multilayer fascial tissue coverage coverage.

### Outcome measures

① Surgery-related indicators: Comparative analysis was performed on the operation time, total intraoperative amount of bleeding, postoperative length of stay in the hospital and other indicators between the two groups; ② Surgical effects between two groups of male children were compared and analyzed according to the following criteria for effect evaluation: (1) significant effective: complete disappearance of symptoms and the ability to urinate by standing by the patients themselves; (2) effective: relief of symptoms and the ability to urinate by standing by the patients themselves after treatment, yet with pain and discomfort; and (3) ineffective: no improvement in the symptoms. The total effective rate was calculated based on the formula of (cases of significant effective + effective)/total cases × 100% (8); ③ Comparative analysis of postoperative conditions: The urinary fistula, urethral stricture and not satisfied with the appearance were compared and analyzed 3 months after surgery. The HOSE scoring system was employed to score according to the location, shape, urine flow, residual chordee of penis, urinary fistula and its complexity (satisfied: ≥14 points) (9); and ④ Changes of urodynamic indexes: Changes of urodynamic parameters such as maximum urinary flow rate (*Q*_max_), mean urinary flow rate (*Q*_avc_), post-void residual urine (PVR) were compared and analyzed before surgery and during the 6-month follow-up period after surgery.

### Statistical analysis

All data were analyzed statistically by SPSS 20.0 software, and the measurement data were expressed by (X¯ ± S). The data between the study group and the control group were analyzed by two-independent sample *t*-test. Moreover, paired *t*-test was used for the comparative analysis of all indicators in the study group before and after treatment, and *χ*^2^ test was used for the comparison of rates. *P* < 0.05 was used to indicate the presence of statistically significant difference.

## Results

A total of 80 children were enrolled in this study, with 40 assigned to the study group and 40 to the control group. Children in the study group were aged between 4 and 11 years old (with an average of 7.20 ± 1.92), and tHOSE in the control group aged between 4 and 10 years old (with an average age of 6.90 ± 1.69). There was no significant difference in the comparison of general data between the two groups (*p* > 0.05), suggesting the comparability between groups (Table 1).

**Table 1:** Comparison of general data in patients between the two groups $(\bar{X}\pm S)n=40$.

Indexes	Study group	Control group	*t*/*χ*^2^	*p*
Age (years)	7.20 ± 1.92	6.90 ± 1.69	0.741	0.461
Urethral orifice			1.003	0.317
Distal (%)	31 (77.50%)	27 (67.50%)		
Central (%)	9 (22.50%)	13 (32.50%)		
Length of urethral defect (cm)	1.70 ± 0.38	1.65 ± 0.42	0.648	0.519
Width of urethral plate (mm)	9.05 ± 1.51	8.97 ± 1.57	0.225	0.823
Combined with mild chordee of penis			0.287	0.592
With (%)	8 (20.00%)	10 (25.00%)		
Without (%)	32 (80.00%)	30 (75.00%)		

*p* > 0.05.

As shown in Table 2, there were statistically significant differences that the operation time (*p* = 0.03) was longer while the postoperative length of stay in the hospital (*p* = 0.000) was shorter in the study group than those in the control group. However, there was no statistical difference in the comparison of the amount of intraoperative bleeding between the study group and the control group (*p* = 0.112).

**Table 2:** Comparison of surgical conditions between the two groups $(\bar{X}\pm S)n=40$.

Groups	Operation time (min)*	Amount of bleeding (mL)	Postoperative length of stay (d)*
Study group	132.58 ± 8.27	16.45 ± 2.86	9.78 ± 1.23
Control group	120.90 ± 8.13	17.55 ± 3.25	12.45 ± 2.06
*t*	6.365	1.608	7.046
*p*	0.000	0.112	0.000

* *p* < 0.05.

According to the comparison of surgical effect between the two groups in Table 3, the effective rate of the study group was 97.50%, while that of the control group was 82.50%. There was statistically significant difference that the effective rate of the former group was obviously higher than that of the latter group (*p* = 0.025).

**Table 3:** Comparison of surgical effect between the two groups $(\bar{X}\pm S)n=40$.

Groups	Significant effective	Effective	Ineffective	Total effective rate*
Study group	37	2	1	39 (97.50%)
Control group	28	5	7	33 (82.50%)
*χ* ^2^				5.000
*p*				0.025

* *p* < 0.05.

In Table 4, the incidence of surgical complications was 7.50% in the study group and 25.00% in the control group 3 months after operation, with a significantly lower rate in the former group than that in the latter group (*p* = 0.034). Meanwhile, there was statistically significant difference that the HOSE score of the study group was significantly higher than that of the control group (*p* = 0.000).

**Table 4:** Comparison of the incidence of complications in the two groups of male children 3 months after operation $(\bar{X}\pm S)n=40$.

Groups	Urinary leakage	Urethral stricture	Not satisfied with the appearance	Incidence of complications*	Hose score
Study group	0	1	2	3 (7.50%)	16.58 ± 1.72
Control group	4	1	5	10 (25.00%)	14.48 ± 1.91
*t*/χ^2^				4.501	5.166
*p*				0.034	0.000

* *p* < 0.05.

There was no significant difference in indicators such as *Q*_max_, *Q*_avc_ and PVR between the two groups before surgery (*p* > 0.05). While the levels of *Q*_max_ and *Q*_avc_ in the study group were higher than those in the control group 6 months after surgery, with statistically significant difference (*p* = 0.000). However, no significant difference was found in postoperative level of PVR between the two groups (*p* = 0.699), (Table 5).

**Table 5:** Comparison of urodynamic parameters before and after operation in the two groups of male children $(\bar{X}\pm S)n=40$.

Indicators	Groups	Study group	Control group	*t*	*p*
*Q*_max_ (mL/s)	Preoperative	8.97 ± 2.02	9.11 ± 1.94	0.310	0.757
	Postoperative*	9.86 ± 1.95	8.04 ± 1.81	4.335	0.000
*Q*_avc_ (mL/s)	Preoperative	6.48 ± 2.31	6.39 ± 1.94	0.194	0.847
	Postoperative*	8.35 ± 2.11	6.08 ± 1.97	4.978	0.000
PVR (mL)	Preoperative	12.66 ± 2.61	12.72 ± 2.34.	0.108	0.914
	Postoperative	10.47 ± 2.58	10.68 ± 2.24	0.389	0.699

* *p* < 0.05.

## Discussion

Hypospadias is a common congenital malformation of the genitourinary tract in male children, accounting for 0.3%–0.5% of newborn boys, and still has an increased incidence recently (10). Among them, distal penile hypospadias accounts for about 70% of all cases of hypospadias (11). As a multifactorial disease, hypospadias may occur owing to the impact of heredity factors, environmental factors, hormone regulation, gene mutations, etc. Considering its complex etiology and pathogenesis, the exact cause of hypospadias can be clarified in only less than 5% of male children (12). Surgery is still the current gold standard for hypospadias (13). The most common complications of surgery for hypospadias are urinary fistula, urethral stricture and unsatisfactory appearance, which brings great psychological pressure to the affected male children and heavy economic burden to their families (14).

Urinary leakage has been estimated to have the highest incidence among all postoperative complications of surgery for hypospadias. Most male children may need to undergo secondary operation or multiple operations owing to the presence of urinary fistula (15). Urinary fistula is generally developed due to the weakness of tissues covering the newly formed urethra or poor blood supply (8), distal urethral obstruction and postoperative infection. Therefore, the innovation and modification of tissue-covering technology has been highly valued for the improvement of surgery for hypospadias in recent decades (16). The tissues to cover the newly established urethra of hypospadias mainly include subcutaneous sarcolemma of penis, tunica vaginalis of testis, Buck's fascia, external spermatic fascia, tunica dartos of the scrotum, etc. (17). For example, Daboos et al. (18) carried out a research based on a random grouping of hypospadias patients into multilayer coverage and no coverage groups. Corresponding results revealed that multilayer coverage could effectively reduce the postoperative complications, especially urinary fistula. It can be considered that coverage of the newly established urethra with multilayer tissue flap can not only reduce the tension of the newly formed urethra, avoid suture overlapping, but also enhance local anti-infection. Meanwhile, by enrolling patients undergoing TIP surgery, Savanelli et al. (19) randomly established two groups of ventral subcutaneous flap coverage group and no coverage group. Consequently, the incidence of urinary fistula in the coverage group was significantly reduced than that in the no coverage group. In addition, according to the report by Mammo et al. (13), the effect of sheath coverage for distal hypospadias was worse than that of vascular pedicle coverage, suggesting the application of double-layer vascular pedicle to cover the newly formed urethra. Moreover, Mammo et al. (13) believed that the newly formed urethra of distal hypospadias was far away from the scrotum, and hence the desired effect was difficult to achieve when there was an excessive stretching of the tissue as it could cause poor tissue blood circulation. Therefore, for the initial treatment of distal hypospadias, it is recommended to cover the newly formed urethra with proximal tissues such as penis sarcolemma, rather than distal tissues (e.g., the tunica vaginalis of testis, tunica dartos of the scrotum, etc.).

Microscopic technology is generally employed under the microscope. Via this approach, tissues and blood vessels can be magnified in the corresponding multiple under the field of vision to provide a clearer surgical field, which can ensure the accuracy of surgery. The technical concept of microsurgery is proposed to ensure the success of operation, which includes fine operation, protection of blood vessels and nerves, adequate protection of normal tissues, and non-invasive suture (20). The key to the success in the surgery for hypospadias is the achievement of tension-free and satisfactory suture of the new urethral lumen. Especially for children with relatively narrow urethral plates, microsurgery has achieved satisfactory anatomical separation of skin flaps, suture of newly formed urethral lumens and coverings under the magnified surgical field by using more sophisticated surgical instruments, thus reducing the occurrence of postoperative complications (21). Significantly, microsurgery for hypospadias has obvious advantages. To be specific, it can reduce the side injury of blood vessels and skin tissues as much as possible, and better protect the blood supply of the pedicled urethral plate flap. Moreover, the anatomical layers of tissues can be clearly distinguished during the shaping and dissociation of skin flaps under microscope to avoid postoperative edema of tissues and urethra, ischemia and necrosis of skin flap, etc. In addition, the use of fine sutures and small suture needles under the microscope for precise suture and smooth anastomotic alignment can accelerate wound healing, smooth wound surface and reduce the risk of stenosis of the newly established urethral lumen, which can eventually reduce postoperative complications and accelerate postoperative healing (22). Besides, as suggested by El-Karamany et al. (23), the application of microsurgery can also reduce postoperative pain, short the length of stay in the hospital and dressing change time, and decrease the incidence of postoperative complications for patients with distal hypospadias.

Furthermore, urethral stricture, second only to urethral fistula, is also one of the common complications after urethroplasty for hypospadias. According to previous report, the incidence of urethral stricture was 10%–20% after surgery for hypospadias (24), which occurred usually at the proximal and distal urethral anastomosis and the distal urethral duct 1–6 months after surgery. Therefore, early identification of urethral stricture and timely treatment are crucial for postoperative recovery and long-term effect of male children (25). Measurement of urinary flow rate is simple and non-invasive to detect urethral stricture (26). Abnormal urinary flow has been considered to have an inevitable relationship with urethral stricture to some extent (27). Abnormal urinary flow is an early warning of the risk of urethral stricture in 50% of cases after surgery for hypospadias. It highlights the necessity of preoperative and postoperative urinary flow measurement. Among them, *Q*_max_ is a simple and effective choice to evaluate urethral stricture (28). There is a reason to suspect urethral stricture when there is a reduced urinary flow rate.

In the present study, the hospital stay of the study group was significantly shorter than that of the control group, which may be related to the minimal trauma of microsurgery and faster postoperative recovery, suggesting that this surgical approach has advantages in accelerating recovery. The effective rate in the study group and the control group was 97.50% and 82.50%, respectively, with statistically significant difference between groups (*p* = 0.025). Meanwhile, comparison of the incidence of surgical complications showed statistically significant difference between the study group (6%) and the control group (20%) 3 months after operation (*p* = 0.034). The HOSE score in the study group was significantly higher than that in the control group, with a statistically significant difference (*p* = 0.000). Besides, statistically significant difference was also observed that the levels of *Q*_max_ and *Q*_avc_ in the study group were higher than those in the control group 6 months after operation (*p* = 0.000).

## Conclusions

To sum up, microsurgery combined with covering new urethra by multilayer fascial tissue coverage tissues has some significance in preventing postoperative complications in male children with hypospadias. It exhibits more significant therapeutic effect, which is manifested in the shorter length of hospital stay, lower incidence of postoperative complications, and significantly improved urodynamic indicators. Findings in our study support the potential clinical application value of this approach in the surgical treatment of male children with hypospadias.

Our study still has some limitation, such as the experimental design of single-center study that may have certain selection bias. The follow-up period of this study was relatively short (6 months), which failed to fully evaluate long-term complications and the sustainability of urodynamic improvements. Future studies will extend the follow-up to 24 months and set multiple time points to assess the therapeutic effects. Our study failed to establish a control group of microsurgery combined with covering new urethra by single-layer sarcolemma tissues. In the future, there is a need for multi-center standardized clinical research based on a larger sample size, so as to objectively evaluate the advantages and disadvantages of this surgical strategy.

## Data Availability

The raw data supporting the conclusions of this article will be made available by the authors, without undue reservation.
